# Pulmonary Consumption

**Published:** 1872-05

**Authors:** 


*Pulmonary Consumption*: its Nature, Varieties, and Treatment. With an analysis of one thousand cases to exemplify its duration. By C. B. J. WILLIAMS, M. D., F. R. S. etc. & CHARLES T. WILLIAMS, M. A., M. D., Oxon. Philadelphia: HENRY C. LEA, 1872.

The large experience of Dr. Williams in the treatment of Pulmonary disorders has enabled him to place in the hands of the profession a work which is in many respects superior to any work of the kind which has fallen under our observation. The labor which was necessary in the arrangement and analysis of the vast number of cases which are spoken of in this work must have been enormous and the profession owe to Dr. Williams and his son many thanks for their untiring zeal and devotion in the cause of Medical science. The chapters on climate may not, and will not meet the wants of the American practitioner in many cases, as he constantly has cases coming under his charge who can not bear the expense of a long voyage to test the climate of the countries spoken of in Dr. Williams' work. Hence, if it had been possible to introduce some additional hints in regard to American climate, which in some localities we believe to be equally efficacious with those of European countries; the work would have more fully met the wants of the American reader. The work nevertheless will be, we doubt not, fully valued and appreciated by all in whose hands it may fall, and is deserving of careful study.

